# Antimicrobial, antioxidant, and sun protection potential of the isolated compounds from *Spermacoce princeae* (K. Schum)

**DOI:** 10.1186/s12906-023-04026-4

**Published:** 2023-06-19

**Authors:** Peter Sekandi, Jane Namukobe, Robert Byamukama, Christine Betty Nagawa, Stefano Barbini, Markus Bacher, Stefan Böhmdorfer, Thomas Rosenau

**Affiliations:** 1 Department of Chemistry, College of Natural Sciences, Makerere University, P.O. Box 7062, Kampala, Uganda; 2 Department of Forestry, Biodiversity, and Tourism, College of Agriculture and Environmental Sciences, Makerere University, P.O. Box 7062, Kampala, Uganda; 3 Institute of Chemistry of Renewable Resources, Department of Chemistry, University of Natural Resources and Life Sciences, Vienna, Vienna, Austria

**Keywords:** Skin infections, *Spermacoce princeae*, Antioxidant activity, Antimicrobial activity, Sun protection potential

## Abstract

**Background:**

*Spermacoce princeae* (K. Schum) has been used in the treatment of bacterial skin infections in Uganda. Pharmacological studies revealed that extracts of *S. princeae* exhibited antibacterial, antioxidant, and sun protection potential. This study aimed at isolating and identifying pure compounds from the extracts based on comprehensive analytical characterization by multiple analytical techniques.

**Methods:**

The plant samples were extracted by sequential maceration using *n-*hexane, ethyl acetate, methanol, and distilled water. The compounds were isolated using a combination of chromatographic techniques and their structures were elucidated by multiple spectroscopic techniques. The antibacterial and antifungal activity determination of the isolated compounds was carried out using an agar well diffusion and potato dextrose assay against *Pseudomonas aeruginosa*, *Staphylococcus aureus, Escherichia coli, Klebsiella pneumoniae, Candida albicans, and Aspergillus flavus* while the antioxidant activity was screened with the 2,2-diphenyl-2-picryl-hydrazyl (DPPH) radical scavenging assay. The sun protection factor was determined using a Shimadzu Ultra Violet-visible (UV–VIS) double beam spectrophotometer between 290 to 320 nm.

**Results:**

Eleven compounds; quercetin (**1**), kaempferol-3-*O*-rutinoside (**2**), rutin (**3**, **12**), *myo-*inositol (**4**), asperulosidic acid (**5**), hexadecanoic acid (**6**), β-sitosterol (**7**), stigmasterol (**8**), campesterol (**9**), ursolic acid (**10**), and β-sitosterol glucoside (**11**) were identified in the *S. princeae* extracts. Compound **2** had good antifungal activity against *C. albicans* (zone of inhibition, 23.0 ± 0.1 mm). Compound **10** showed antibacterial and antifungal activity against *S*. *aureus, P. aeruginosa, C. albicans, and A. flavus*. Compound **2** had a good percentage radical scavenging effect (IC_50_ = 64.81 µg/ml) and a good sun protection factor (SPF = 26.83).

**Conclusion:**

This study reports the first-time isolation and identification of compounds **1** to **11** from *S. princeae,* which contribute to its antimicrobial, antioxidant, and sun protection potential*.*

## Introduction

*Spermacoce princeae* from the genus Spermacoce is an annual flowering herb endemic to tropical Asia, Africa, and East India [1, 2]. Spermacoce is a genus of the family Rubiaceae comprising about 275 species. The plants have fimbriate stipules connected to the petioles, with white flowers at maturity arranged in compact lateral inflorescences [3–5]. Species from the genus have been reported to possess medicinal properties. For example; the seeds of *Spermacoce hispida* in India alleviate liver and kidney damage associated with oxidative stress [6], *S. princeae* in Kenya and Cameroon has been used to treat bacterial infection [3]. *S. princeae* is locally known as “Ekaiza nkoju” in the Kiswahili language [7]. In Uganda, the plant is known as either “musanvuma/enkokoma enkazi” in Luganda or “Kisakimu” in the Rutoro dialect [8]. Aqueous extracts from leaves and roots are used for the management and treatment of malaria, cancer, wounds, eye and skin diseases, among others [3, 7, 9]. Water extracts of *S. princeae* fresh leaves are taken orally by pregnant women to induce labor during childbirth or are applied on skin cuts to treat wounds. In Central and Eastern Uganda, dry leaves are pounded, mixed with oil, and smeared on the skin to treat skin infections [10]. Previous phytochemical screening of *S. princeae* extracts revealeds the presence of saponins, alkaloids, glycosides, tannins, flavonoids, and terpenoids [1, 2]. Our previous pharmacological study showed that *S. princeae* extracts (MeOH and water) were active against *S. aureus*, *K. pneumoniae,* and *P. aeruginosa*. The same study revealed that the methanol and aqueous extracts exhibited good antioxidant activity [11]. Some species of the genera Spermacoce have been studied and more than 60 compounds from different compound classes have been isolated. For example; stigmasterol, benzo-isoquinoline, and sitostenone among others have been isolated from *S. exilis*, *S. verticillate,* and *S. articularis* [12, 13]. There is no report on the active compounds from *S. princeae* (K. Schum) and their isolation. The purpose of this paper is to isolate and determine active compounds from *S. princeae* extracts and to study their antibacterial, antifungal, antioxidant, and sun protection activities.

## Materials and methods

### Sample collection and preparation

**Fig. 1:**
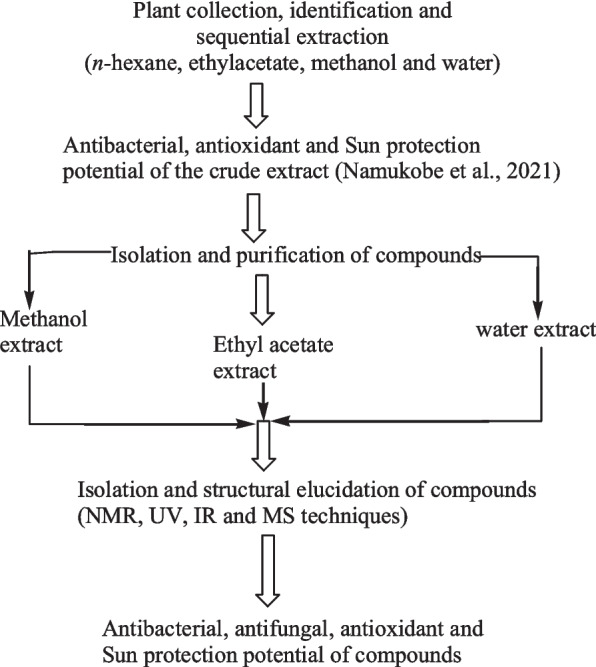
Flow chart of the procedures carried out in the study

Plant collection and extraction were carried out as previously reported [11]. After identification and authentication (Mr. Rwaburindore Portase, Makerere University Herbarium, Department of Botany), the leaves of *S. princeace* were collected along the shores of Ndura water stream, 2 km from the Makerere University Biological field station, Fort portal. The plant sample was collected with the assistance of local leaders and indigenous people after obtaining permission from National Forestry Authority. A Voucher specimen number, 002 in account number 50892, has been deposited at the Makerere University Herbarium, College of Natural Sciences, Department of Plant Science, Microbiology, and Biotechnology for future reference. The samples were air-dried at room temperature for 28 days. The dry samples were then ground into a fine powder. The powders were then sealed in air-tight polythene bags and stored in a cool dry place. The powdered sample (1.0 kg) was extracted sequentially by maceration using *n-*hexane, ethyl acetate, methanol, and distilled water. The extraction was carried out five times using 3 L of solvent at each time. The extracts were filtered through cotton fabric followed by Whatman No.1 filter paper and concentrated using a rotary evaporator (Buchi, R300) at 40 °C to dryness. The dried extracts were transferred to sample bottles which were placed in a desiccator containing anhydrous sodium sulphate to remove any traces of water. The dried extracts were later put in tightly stoppered sample bottles and stored in a refrigerator. Figure 1 shows the flow chart of the experimental procedures of the study. Sequential extraction allows a set of phytochemicals to be extracted according to polarity, starting with apolar substances, such as essentiaol oils, going to polar compounds such as flavonoids [14].

### Isolation and purification of compounds from extracts

The methanol extract (16.2 g) was subjected to column chromatography using a gradient solvent system of *n-*hexane/ethyl acetate (EtOAc) and EtOAc/methanol (MeOH) affording 12 fractions (M_1-12_), after monitoring separation using analytical thin layer chromatography (TLC) on aluminum plates precoated with silica gel. The TLC plates were used to develop the solvent system used in the purification of the compounds [15–17]. Fraction M_3_ (0.313 g) was subjected to column chromatography using a gradient solvent system of *n-*hexane/EtOAc (from 7:3 to 1:1, v/v), and EtOAc/MeOH (95:5, v/v) to obtain 16 sub-fractions (C_1_-_16_). Sub-fraction C_16_ was purified on Sephadex LH-20 with CHCl_3_/MeOH (1:1, v/v) to obtain compound **1** (12 mg). Fraction M_9_ (1.693 g) was subjected to column chromatography on silica gel with *n-*hexane/EtOAc to obtain 13 subfractions (P_1-13_). Sub-fraction P_8_ was purified on silica gel using *n-*hexane/EtOAc (75:25, v/v) to obtain compound **2** (14.2 mg). Fraction M_10_ (5.398 g) was subjected to column chromatography using a gradient solvent system of EtOAc/MeOH to yield 9 subfractions (J_1_-_9_). Subfraction J_4_ was purified on a silica gel column using EtOAc/tert butanol/H_2_O (65:25:9, v/v/v) to obtain compound **3** (15 mg) [18]. Compound **4** (5 mg) which crystallized out of subfraction J_7,_ was filtered off, and washed with pure MeOH. Fraction M_11_ (2.921 g) was purified on silica gel with EtOAc/MeOH/H_2_O (20:3:2, v/v/v) to obtain 14 subfractions (N_1-14_). Subfraction N_7_ was subjected to repeated column chromatography with EtOAc/MeOH/H_2_O (20: 3: 2 v/v/v) to yield compound **5** (5 mg) [19].

The EtOAc extract (20.253 g) was subjected to silica gel column chromatography with *n-*hexane/EtOAc and EtOAc/MeOH affording 21 fractions (E_1_-_21_) [20, 21]. Fraction E_5_ was subjected to repeated column chromatography on silica gel with *n-*hexane/CH_2_Cl_2_ (1:1, v/v) to obtain compound **6** (15.1 mg) and fraction E_6_ (0.343 g) with *n-*hexane/CH_2_Cl_2_ (4:1, v/v) to obtain 24 subfractions (E6_(1–24)_). Subfraction E-_6_–_3_ precipitated needle-like crystals, which were washed with pure hexane to obtain a mixture of compounds **7**, **8**, and **9** (10.0 mg). Fraction E_13_ (1.021 g) was washed with pure EtOAc followed by pure MeOH. The MeOH filtrate (E_13m_) was subjected to silica gel column chromatography using acetonitrile (MeCN) to obtain compound **10** (23 mg). Fraction E_18_ (0.803 g) was subjected to repeated column chromatography on silica gel using *n-*hexane/EtOAc (100:30, v/v) to obtain compound **11** (5.7 mg).

The aqueous extract (76 g) was partitioned in CH_2_Cl_2_/H_2_O (1:1, v/v) in a separating funnel [18, 22]. The mixture was shaken for 10 min and left for phase separation. The organic layer was collected and evaporated on a rotary evaporator at 40 ^0^C up to dryness. The organic extract (70.5 mg) was subjected to silica gel column chromatography using *n-*hexane/EtOAc (1:1, v/v) affording 6 subfractions (AO_1-6_). Subfraction AO_6_ (10 mg) was purified using preparative TLC with a solvent system of EtOAc/*tert*-butanol/H_2_O/acetic acid (20:3:1:1, v/v/v/v) to obtain compound **12** (4.9 mg).

### Spectroscopic analysis of the isolated compounds

The Fourier transform infrared (FT-IR) and UV/VIS spectra of isolated compounds were recorded on a PerkinElmer FT-IR and double-beam UV/VIS Frontier spectrophotometer respectively [23]. All nuclear magnetic resonance (NMR) spectra were recorded on a Bruker Avance II 400 MHz instrument (resonance frequencies 400.13 MHz for ^1^H and 100.61 MHz for ^13^C) equipped with a 5 mm N_2_-cooled broadband cryo-probe-head (Prodigy) with z–gradients at room temperature with standard Bruker pulse programs. The samples were dissolved in 0.6 ml of either CDCl_3_, DMSO-d_6_, MeO-d_4_, or D_2_O (all Eurisotop, Saint-Aubin, France). Chemical shifts are given in ppm, referenced to residual solvent signals (CDCl_3_: δ_H_/δ_C_ 7.26 / 77.0 ppm, DMSO-d_6_: δ_H_/δ_C_ 2.49 / 39.6 ppm, MeO-d_4_: δ_H_/δ_C_ 3.31 / 49.0 ppm) or in the case of D_2_O by addition of one drop of acetone δ_H_/δ_C_ 2.22 / 30.9 ppm) [24]. Ultra-Performance Convergence Chromatography Quadrupole Time-of-Flight Mass Spectrometry (UPC^2^-QTof-MS) was used to support the structural assignment of the compounds [25, 26]. The structures of the compounds were identified by interpretation of their spectral data and by comparison with those reported in the literature.

### GC–MS/FID analysis

Gas Chromatography (Agilent Technologies 5975C) coupled to mass spectrometry (MSD inert XL TAD) and a flame ionization detector (FID) were used to analyze subfraction E-6–3 from which the MS of compounds **7**, **8**, and **9** were recorded. The MS detector was operated in the electron-impact (EI) mode at 70 eV using a temperature of 280 °C. The mass scanning range was set to 29–1050 amu (atomic mass unit), and the solvent cutting time was 4 min. The FID was operated at 400 °C, with H_2_ flow of 30 mL/ min, air flow of 400 mL/ min, and makeup flow (combined) of 25 mL/ min. The GC device was fitted with a UltiMetal VF-5ht capillary column (30 m × 250 µm × 0.10 µm, Agilent J&W). The column temperature program was set as follows: initial T = 65 °C isothermal for 5 min, ramp to 380 °C (rate, 10 °C/ min), and maintain at 380 °C for 8 min. Helium was used as a carrier gas, with a gas flow of 2.5 mL/ min. Injection (1.0 μL) was performed by an autosampler in a cold multimode inlet (MMI), which was kept at 65 °C for 6 s, increased to 380 °C at 500 °C/ min, and then held for 5 min (cold split injection). The split ratio was set to 15:1 (split flow, 37.5 mL/ min). The total analysis time was 44.50 min. Compounds were analyzed with GC as their trimethylsilyl derivatives. 200 μL of silylating agent composed of 9:1, v/v of *N,N*-bis(trimethylsilyl)-trifluoroacetamide (BSTFA, ≥ 99%, Sigma-Aldrich) and trimethylchlorosilane (TMCS, ≥ 99%, Sigma-Aldrich) respectively were added to each vial, which contained 5 mg of homogenized sample [27–30]. Five drops of hydrous pyridine (99.8%, Sigma-Aldrich) were also added to each vial before the vortex. All spectra were analyzed with Enhanced ChemStation (MSD ChemStation F.01.01.2317), deconvoluted, and then evaluated using the Mass Hunter Workstation software. The compounds were identified by comparison with Wiley10 and the National Institute of Standard and Technology (NIST17) mass spectral library.

### Spectroscopic data of the isolated compounds

Quercetin (**1**) [31, 32]: yellow powder: FT-IR: 3214, 2924, 1652, 1598, 1504, 1441, 1366, 1259, 1163, 1089 and 1008 cm^−1^: UV λ_max_ MeOH (nm); 255 and 370: ^1^H-NMR (400 MHz, CD_3_OD), δ_H_ 7.73 (H-2’, *d, J* = *2.1* Hz, 1H), 7.63 (H-6’, *dd, J* = *2.1, 8.5* Hz, 1H)*,* 6.88 (H-5’, *d, J* = *8.5* Hz, 1H), 6.39 (H-8, *d, J* = *2.0* Hz, 1H) and 6.18, (H-6, *d, J* = *2.0* Hz, 1H); ^13^C-NMR (100 MHz, CD_3_OD): δ_C_ 177.4 (C-4), 165.7 (C-7), 162.6 (C-5), 158.3 (C-9), 148.8 (C-4’), 148.1 (C-2), 146.3 (C-3’), 137.2 (C-3), 124.3 (C-1’), 121.7 (C-6’), 116.3 (C-5’), 115.9 (C-2’), 104.5 (C-10), 99.2 (C-6) and 94.3 (C-8): UPC^2^-QTof-MS (positive mode) *m/z* 341.0058 [M + K]^+^, C_15_H_10_O_7._

Kaempferol-3-*O*-rutinoside (**2**) [33, 34]: yellow powder: FT-IR: 3285, 2916, 1652, 1598, 1498, 1359, 1179 and 1065 cm^−1^_:_ UV λ_max_ MeOH (nm); 266 and 349: ^1^H-NMR (400 MHz, CD_3_OD), δ_H_ 8.07 (H-2’/6’, d*, J* = *8.9* Hz, 2H), 6.89 (H-3’/5’, d*, J* = *8.9* Hz, 2H), 6.42 (H-8, *d, J* = *2.0* Hz, 1H), 6.21 (H-6, *d, J* = *2.0* Hz, 1H), 5.12 (H-1’’, *d, J* = 7.6 Hz, 1H), 4.51 (H-1^’’’^, *d, J* = *1.5* Hz, 1H), 3.81 (H-6 ‘‘a, *d, J* = 10.5 Hz, 1H), 3.63 (H-2 ‘‘‘, *dd, J* = 3.3, 1.5 Hz, 1H), 3.52 (H-3 ‘‘‘, *dd, J* = *9.4, 3.3 Hz,* 1H), 3.45 (H-5 ‘‘‘, *m,* 1H), 3.44 (H-2 ‘‘, *m,* 1H), 3.41 (H-3 ‘‘, *m,* 1H), 3.37 (H-6 ‘‘b, *m*, 1H), 3.33 (H-5 ‘‘, *m,* 1H), 3.28 (H-4 ‘‘‘, *m,* 1H), 3.25 (H-4 ‘‘, *m,* 1H), and 1.12 (H-6 ‘‘‘, *d, J* = *6.2* Hz, 3H); ^13^C-NMR (100 MHz, CD_3_OD), δ_C_ 179.4 (C-4), 166.2 (C-7), 163.1 (C-5), 161.5 (C-4’),159.6 (C-2), 158.5 (C-9), 135.6 (C-3), 132.4 (C-2’/6 ‘) 122.7 (C-1’), 116.2 (C-3’/5 ‘), 105.6 (C-10), 104.7 (C-1’’), 102.4 (C-1’’’), 100.1 (C-6), 95.0 (C-8), 78.1 (C-3’’), 77.2 (C-5’’), 75.7 (C-2’’), 74.0 (C-4’’’), 72.4 (C-3’’’), 72.2 (C-2’’’), 71.4 (C-4’’), 69.7 (C-5’’’), 68.5 (C-6’’), 18.0 (C-6’’’): UPC^2^-QTof-MS (negative mode) *m/z* 593.1529 [M-H]^−^, C_27_H_30_O_15_.

Rutin (**3**, **12**) [35, 36]: yellow powder: FT-IR; 3332, 2941, 2537, 1646, 1593, 1497, 1452, 1356, 1284, 1202, 1059, 999, 965 and 941 cm^−1^: UV λ_max_ (nm); 257 and 358 nm: ^1^H-NMR (400 MHz, CD_3_OD), δ_H_ 7.66 (H-2’, *d, J* = *2.1* Hz, 1H), 7.63 (H-6’, dd*, J* = *8.4, 2.1* Hz, 1H), 6.87 (H-5’, *d, J* = *8.4* Hz, 1H), 6.40 (H-8, *d, J* = *2.0* Hz, 1H), 6.21 (H-6, *d, J* = *2.0* Hz, 1H), 5.11 (H-1’’, *d, J* = *7.6* Hz, 1H), 4.52 (H-1^’’’^, *d, J* = *1.5* Hz, 1H), 3.81 (H-6 ‘‘a, *dd, J* = *10.9, 1.2* Hz, 1H), 3.63 (H-2 ‘‘‘, *dd, J* = *3.4, 1.7* Hz, 1H), 3.53 (H-3 ‘‘‘, *dd, J* = *9.5, 3.4* Hz, 1H), 3.46 (H-2 ‘‘, *m,* 1H), 3.45 (H-5 ‘‘‘, *m,* 1H), 3.41 (H-3 ‘‘, *m,* 1H), 3.39 (H-6 ‘‘b, m, 1H), 3.32 (H-5 ‘‘, *m,* 1H), 3.28 (H-4 ‘‘‘, *m,* 1H), 3.26 (H-4 ‘‘, *m,* 1H), 1.12 (H-6 ‘‘‘, *d, J* = *6.2* Hz, 3H). ^13^C-NMR (100 MHz, CD_3_OD); δ_C_ 179.4 (C-4), 166.1 (C-7), 163.0 (C-5), 159.3 (C-2), 158.5 (C-9), 149.8 (C-4’), 145.8 (C-3’), 135.6 (C-3), 123.5 (C-6’), 123.1 (C-1’), 117.7 (C-2’), 116.1 (C-5’), 105.6 (C-10), 104.7 (C-1’’), 102.4 (C-1’’’), 99.9 (C-6), 94.8 (C-8), 78.3 (C-3’’), 77.2 (C-5’’), 75.7 (C-2’’), 73.9 (C-4’’’), 72.2 (C-3’’’), 72.1 (C-2’’’), 71.4 (C-4’’), 69.6 (C-5’’’), 68.5 (C-6’’), 17.9 (C-6’’’): UPC^2^-QTof-MS (positive mode) *m/z* 633.1426 [M + Na]^+^, C_27_H_30_O_16_.

*Myo-*inositol (**4**) [37]: white crystalline solid: FT-IR: 3304, 1634, 1408 and 1050 cm^−1^: ^1^H-NMR (400 MHz, D_2_O), δ_H_ 4.04 (H-4, *t, J* = *2.8* Hz 1H), 3.61 (H-2/6, *dd, J* = *10.0, 9.4* Hz, 2H), 3.52 (H-3/5, *dd, J* = 10.0, 2.8 Hz, 2H), 3.26 (H-1, *t, J* = *9.4* Hz, 1H); ^13^C-NMR (100 MHz, CD_3_OD): δ_C_ 75.0 (C-1), 73.0 (C-2,6), 72.8 (C-4), 71.8 (C-3,5): UPC^2^-QTof-MS (positive mode) *m/z* 203.0526 [M + Na]^+^, C_6_H_12_O_6_.

Asperulosidic acid (**5**) [38]: white solid: FT-IR: 3339, 2902, 1578, 1410, 1250, 1075, 1029 and 931 cm^−1^: UV λ_max_ MeOH (nm); 252: ^1^H-NMR (400 MHz, CD_3_OD), 7.42 (H-3, *s*, 1H), 5.98 (H-7, *s*, 1H), 4.97 (H-1, *d, J* = *8.8* Hz, 1H), 4.94 (H-10a, d, *J* = 15 Hz, 1H), 4.89 (H-6, *m*, 1H), 4.81 (H-10b, d, *J* = 15 Hz, 1H), 4.71 (H-1’, *d, J* = *7.8* Hz, 1H), 3.84 (H-6’a, *dd, J* = *12.3, 1.6* Hz, 2H), 3.62 (H-6’b, dd, *J* = 12.3, 6.0 Hz, 1H), 3.38 (H-3’, *m,* 1H), 3.26 (H-4’,H-5’ *m*, 2H), 3.23 (H-2’, *dd, J* = *9.1, 7.8* Hz*,* 1H), 3.05 (H-5, *br.t, J* = *3.1,* 1H), 2.59 (H-9, *pseudo*-*t,* J = 8.2 Hz, 1H), 2.09 (H-12, *s*, 3H); ^13^C-NMR (100 MHz, CD_3_OD): δ_C_ 172.6 (C-11), 170.2 (C-13), 151.6 (C-3), 146.2 (C-8), 131.8 (C-7), 113.7 (C-4), 100.7 (C-1), 100.4 (C-1 ‘), 78.6 (C-5’), 78.0 (C-3’), 76.0 (C-6), 75.1 (C-2’), 71.8 (C-3’), 64.1 (C-10), 63.1 (C-6’), 47.0 (C-9), 43.7 (C-5), 20.9 (C-12). UPC^2^-QTof-MS (negative mode) *m/z* 431.1186 [M-H]^−^, C_18_H_24_O_12_.

Hexadecanoic acid (**6**) [39]: oily liquid: FTIR: 3380, 2955, 2915, 2848, 1698, 1464, 1464, 1292 and 940 cm^−1^: ^1^H NMR (400 MHz, CDCl_3_), δ_H_ 2.34 (H-2, *t,* 2H), 1.62 (H-3, *p*, 2H), 1.28 (H-15, *m,* 2H), 1.25 (H-4,5,6,7,8,9,10,11,12, *m,* 2H) and 0.88 (3H, *t,* H-16); ^13^C NMR (100 MHz, CDCl_3_): δ_C_ 180.0 (C-1), 34.2 (C-2), 32.2 (C-14), 29.9 (C-10–13), 29.8 (C-5/6), 29.6 (C-4), 29.5 (C-8), 29.4 (C-9), 29.2 (C-7), 24.9 (C-3), 22.9 (C-15), and 14.3 (C-16): UPC^2^-QTof-MS (negative mode) *m/z* 255.2329 [M-H]^−^; C_16_H_32_O_2._

β-Sitosterol (**7**) [21, 40]: white needle-like crystals: FT-IR: 3329, 2933, 2866, 1689, 1456, 1374, 1192, 1052, 960 and 838 cm^−1^: ^1^H-NMR (400 MHz, CDCl_3_), δ_H_ 5.35 (H-6, *m*, 1H), 3.52 (H-3, *m*, 1H), 2.30 (H-4a*, ddd*, *J* = 13.1, 5.1, 1.9 Hz), 2.25 (H-4b*, dm*, *J* = 13.1 Hz), 2.01 (H-12a*, m*, 1H), 1.98 (H-7a*, m*, 1H), 1.85 (H-1a*, m*, 1H), 1.84 (H-2a, H-16a*, m*, 2H), 1.67 (H-25, *m*, 1H), 1.58 (H-15a*, m*, 1H,), 1.54 (H-7b*, m*, 1H), 1.51 (H-2b*, m*, 1H), 1.50 (H-11a, *m*, 1H), 1.46 (H-11b*, m*, 1H), 1.45 (H-8, *m*, 1H), 1.36 (H-20, *m*, 1H), 1.33 (H-22a*, m*, 1H), 1.27 (H-16b*, m*, 1H), 1.25 (H-24^1^, *m* 2H,), 1.17 (H-23, *m*, 1H), 1.16 (H-12b*, m*, 1H), 1.12 (H-17, *m*, 1H), 1.08 (H-1b*, m*, 1H), 1.07 (H-15b*, m*, 1H), 1.02 (H-22b*, m*, 1H), 1.01 (H-19, *s*, 3H), 1.00 (H-14, *m*, 1H), 0.93 (H-9, H-24, *m*, 2H), 0.92 (H-21, *d*, *J* = *6.7* Hz, 3H), 0.85 (H-24^2^*, t, J* = *7.4* Hz, 3H), 0.84 (H-27, *d*, *J* = 7.5 Hz, 3H), 0.82 (H-26, *d*, *J* = *6.9* Hz, 3H), 0.68 (H-18, *s*, 3H);

^13^C-NMR (CDCl_3_) 140.8 (C-5), 121.7 (C-6), 71.8 (C-3), 56.8 (C-14), 56.1 (C-17), 50.2 (C-9), 45.9 (C-24), 42.3 (C-4, C-13), 39.8 (C-12), 37.3 (C-1), 36.5 (C-10), 36.2 (C-20), 34.0 (C-22), 31.93 (C-8), 31.91 (C-7), 31.7 (C-2), 29.2 (C-25), 28.2 (C-16), 26.1 (C-23), 24.3 (C-15), 23.1 (C-24^1^), 21.1 (C-11), 19.8 (C-27), 19.4 (C-19), 19.0 (C-26), 18.3 (C-21), 12.0 (C-24^2^), 11.9 (C-18): GC–MS molecular mass 413 ${[\mathrm{M}]}^{+.}$,calculated for C_29_H_49_O.

Stigmasterol (**8**) [41]: white needle-like crystals: FT-IR (see FT-IR of compound 7): ^1^H-NMR (400 MHz, CDCl_3_), δ_H_ 5.35 (H-6, *m*, 1H), 5.16 (H-22, *dd*, *J* = 15.2, 8.5 Hz, 1H), 5.02 (H-23, *dd*, *J* = 15.2, 8.5 Hz, 1H), 3.52 (H-3, *m*, 1H), 2.30 (H-4a*, ddd*, *J* = 13.1, 5.1, 1.9 Hz), 2.25 (H-4b*, dm*, *J* = 13.1 Hz), 2.05 (H-20, *m*, 1H), 2.01 (H-12a*, m*, 1H), 1.98 (H-7a*, m*, 1H), 1.85 (H-1a*, m*, 1H), 1.84 (H-2a, H-16a*, m*, 2H), 1.67 (H-25, *m*, 1H), 1.58 (H-15a*, m*, 1H), 1.54 (H-7b*,* H-24,* m*, 2H), 1.51 (H-2b*, m*, 1H), 1.50 (H-11a, *m*, 1H), 1.46 (H-11b*, m*, 1H), 1.45 (H-8, *m*, 1H), 1.27 (H-16b*, m*, 1H), 1.25 (H-24^1^, *m* 2H,), 1.16 (H-12b*, m*, 1H), 1.12 (H-17, *m*, 1H), 1.08 (H-1b*, m*, 1H), 1.07 (H-15b*, m*, 1H), 1.02 (H-21, *d*, *J* = 6.5 Hz, 3H), 1.01 (H-19, *s*, 3H), 1.00 (H-14, *m*, 1H), 0.93 (H-9, H-24, *m*, 2H), 0.85 (H-24^2^*, t, J* = *7.4* Hz, 3H), 0.84 (H-27, *d*, *J* = 7.5 Hz, 3H), 0.82 (H-26, *d*, *J* = *6.9* Hz, 3H), 0.68 (H-18, *s*, 3H);

^13^C-NMR (CDCl_3_) 140.8 (C-5), 138.3 (C-22), 129.3 (C-23), 121.7 (C-6), 71.8 (C-3), 56.8 (C-14), 56.1 (C-17), 51.2 (C-24), 50.2 (C-9), 42.3 (C-4, C-13), 40.5 (C-20), 39.8 (C-12), 37.3 (C-1), 36.5 (C-10), 31.93 (C-8), 31.91 (C-7), 31.7 (C-2), 29.2 (C-25), 28.2 (C-16), 24.3 (C-15), 23.1 (C-24^1^), 21.1 (C-11), 21.2 (C-21), 19.8 (C-27), 19.4 (C-19), 19.0 (C-26), 12.0 (C-24^2^), 11.9 (C-18); GC–MS molecular mass 411 ${[\mathrm{M}]}^{+.}$, calculated for C_29_H_47_O.

Campesterol (**9**): white need-like crystals: FT-IR (see FT-IR of compound 7): RT, 27.2 min: GC–MS molecular mass 399 ${[\mathrm{M}]}^{+.}$, calculated for C_28_H_47_O.

Ursolic acid (**10**) [42, 43]: white solids: FT-IR: 3379, 2919, 1687, 1454, 1372, 1162, 1035 and 800 cm^−1^: ^1^H -NMR (400 MHz, DMSO-d6), δ_H_ 5.10 (br.t, *J* = 3.3, H-12), 2.98 (dd, *J* = 10.2, 5.6, H-3), 2.10 (d, *J* = 11.3, H-18), 1.90 (m, H-16a), 1.83 (m, H-11a + b), 1.82 (m, H-15a), 1.52 (m, H-22a + b), 1.51 (m, H-1a, H-16b), 1.46 (m, H-6a), 1.44 (m, H2a + b, H-9), 1.42 (m, H-7a, H-21a), 1.29 (m, H-6b, H-19), 1.26 (m, H-21b), 1.25 (m, H-7b), 1.02 (s, H-27), 0.97 (m, H-15b), 0.92 (m, H-20), 0.90 (m, H-1b), 0.89 (d, *J* = 6.6, H-29), 0.88 (s, H-23), 0.85 (s, H-25), 0.80 (d, *J* = 6.4, H-30), 0.74 (s, H-26), 0–66 (m, H-5), 0.66 (s, H-24); ^13^C -NMR (100 MHz, DMSO-d6), δc 178.4 (C-28), 138.3 (C-13), 124.4 (C-12), 76.8 (C-3), 54.8 (C-5), 52.4 (C-18), 47.0 (C- 9), 46.8 (C-17), 41.8 (C-14), 39.2 (C-8), 38.62 (C-20), 38.56 (C-19), 38.5 (C-4), 38.2 (C-1), 36.6 (C-22), 36.5 (C-10), 32.7 (C-7), 30.2 (C-21), 28.2 (C-23), 27.5 (C-15), 27.0 (C-2), 23.8 (C-16), 23.2 (C-27), 22.8 (C-11), 21.1 (C-29), 18.0 (C-6), 17.14 (C-30), 17.06 (C-26), 16.0 (C-24), 15.2 (C-25); UPC^2^-QTof-MS *m/z* 455.3518 [M-H]^+^, C_30_H_48_O_3_.

β-Sitosterol glucoside (**11**) [20, 44]: white solids: FT-IR: 3380, 2935, 2868, 1454, 1372, 1254, 1162, 1035, 925 and 799 cm^−1^: ^1^H -NMR (400 MHz, DMSO-d6), δ_H_ 5.31 (m, H-6), 4.89 (d, *J* = 4.8, 3’-OH), 4.87 (d, *J* = 4.8, 2’-OH), 4.86 (d, *J* = 4.7, 4’-OH), 4.43 (t, *J* = 6.0, 6’-OH), 4.20 (d, *J* = 7.7, H-1’), 3.63 (dd, *J* = 11.7, 5.5, H-6’a), 3.39 (m, H-6’b), 3.45 (m, H-3), 3.10 (m, H-3’), 3.08 (m, H-5’), 3.00 (m, H-4’), 2.88 (ddd, *J* = 8.6, 7.7, 4.8, H-2’), 2.35 (br.dd, *J* = 13.6, 3.4, H-4a), 2.11 (*pseudo*-t, *J* = 13.6, H-4b), 1.95 (m, H-12a), 1.91 (m, H-7a), 1.80 (m, H-2a), 1.78 (m, H-1a, H-16a), 1.62 (m, H-25), 1.53 (m, H-15a), 1.49 (m, H-7b), 1.47 (m, H-2b), 1.46 (m, H-11a), 1.39 (m, H-11b), 1.38 (m, H-8), 1.32 (m, H-20), 1.29 (m, H-22a), 1.22 (m, H-16b, H-28a + b), 1.13 (m, H-12b, H-23a + b), 1.09 (m, H-17), 1.03 (m, H-15b), 0.99 (m, H-22b), 0.97 (m, H-1b, H-14), 0.94 (s, H-19), 0.90 (m, H-9, H-24), 0.89 (d, *J* = 6.6, H-21), 0.81 (t, *J* = 7.1, H-29), 0.80 (d, *J* = 6.7, H-27), 0.78 (d, *J* = 6.7, H-26), 0.64 (s, H-18); ^13^C -NMR (100 MHz, DMSO-d6), δc 140.4 (C-5), 121.2 (C-6), 100.9 (C-1’), 77.0 (C-3), 76.9 (C-3’, C-5’), 73.6 (C-2′9, 70.2 (C-4’), 61.2 (C-6’), 56.2 (C-14), 55.4 (C-17), 49.6 (C- 9), 45.1 (C-24), 41.8 (C-13), 39.3 (C-12), 38.3 (C-4), 36.8 (C-1), 36.2 (C-10), 35.5 (C-20), 33.3 (C-22), 31.53 (C-8), 31.48 (C-7), 29.3 (C-2), 28.7 (C-25), 27.8 (C-16), 25.4 (C-23), 23.9 (C-15), 22.6 (C-28), 20.6 (C-11), 19.7 (C-27), 19.1 (C-19), 18.9 (C-26), 18.6 (C-21), 11.8 (C-29), 11.7 (C-18).

### Antibacterial and antifungal screening of the isolated compounds

The antibacterial activity of the isolated compounds was investigated according to the agar well diffusion method [45, 46]. Muller-Hinton agar was used for bacterial growth. The inoculum was a culture of each bacterial species in 10 ml of Muller Hinton agar diluted in the same medium to a final concentration of 1 × 10^3^ CFU/ml (0.5 NTU – McFarland scale). Wells were made using a 6 mm diameter of sterile cork borer. For antibacterial screening, the tested compound (10 mg/ml), ciprofloxacin (100 μl), and DMSO as negative control were added to each well separately. The plates were incubated at 37 °C for 24 h. Antifungal tests were carried out using 100 µl of suspension containing a culture of fungi on potato dextrose agar (PDA) incubated at room temperature for 72 h. The antimicrobial activity of the compounds was determined by measuring the diameter of the clear zone around the well. Three replicates were carried out for each experiment [47].

### Antioxidant activity of the isolated compounds

The antioxidant activity of the isolated compounds was determined by a 2,2-diphenyl-1-picryl-hydrazyl (DPPH) assay [48]. A 0.5 mM DPPH solution was prepared by dissolving 19.7 mg of DPPH in 100 ml of distilled methanol and kept in the dark for 45 min at room temperature. Methanoic solution of the isolated compounds and of ascorbic acid as a standard were prepared (2.0 mg/ml each) and diluted to lower concentrations (1000, 500, 250, 125, 62.5 µg/ml). The prepared solutions and DPPH (2000 µl each) were mixed in a cuvette and kept in the dark for 15 min to stabilize. The absorbance of the mixture was measured at 517 nm on a Shimadzu UV–VIS double-beam spectrophotometer against a blank. The concentration of the compound (antioxidant) required to decrease the initial DPPH concentration by 50% (IC_50_) was calculated using Logit regression analysis. A lower IC_50_ value corresponded to a larger scavenging power. All experiments were performed in triplicate and values were expressed as mean ± standard deviation (SD).

### Sun protection potential of the isolated compounds

The sun protection factor was determined according to a modified method reported by Dutra et al. (2004). The compounds were dissolved in methanol without ultra-sonication to a concentration 2 mg/ml. The absorption data of each sample was measured on a JENWAY UV–VIS single beam spectrophotometer between 290 to 320 nm every 5 nm, and methanol as a blank. Para amino-benzoic acid was used as a standard sunscreen. Four measurements were averaged and the sun protection factor was determined using the Mansur equation [49].

### Data analysis

All data were analyzed using descriptive statistics as implemented by Microsoft Excel. The results were generally expressed as mean ± standard deviation (SD).

## Results

**Fig. 2:**
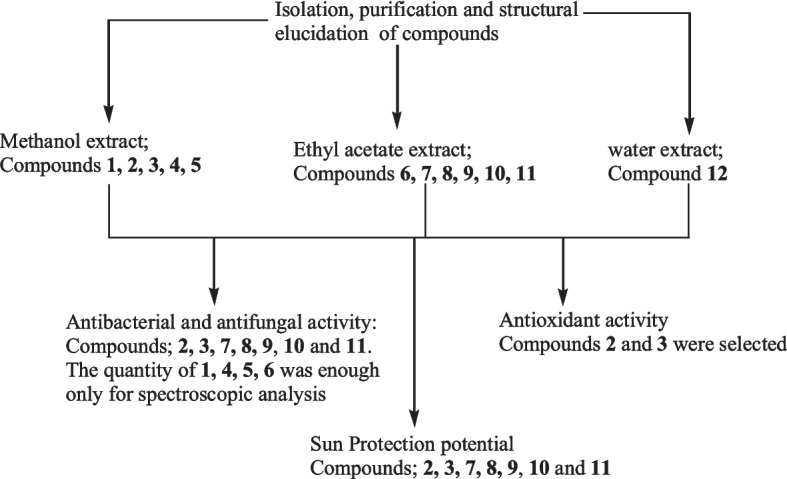
Flow chart showing the isolation and bioactivity testing of the compounds

Eleven bioactive compounds were isolated from the extracts (Fig. 2), namely; quercetin (**1**), kaempferol-3-*O*-rutinoside (**2**), rutin (**3**), *myo-*inositol (4), asperulosidic acid (**5**), hexadecanoic acid (**6**), β-sitosterol (**7**), stigmasterol (**8**), campesterol (**9**), ursolic acid (**10**), and β-sitosterol glucoside (**11**). All compounds were comprehensively analytically characterized and the data compared to literature values. Compounds **1**, **2**, **3**, **4**, and **5** were isolated from the methanolic extract. Compounds **6**, **7**, **8**, **9**, **10**, and **11** from the ethyl acetate extract and compound **12** from the aqueous extract. Figure 2 shows the flow chart of the isolation and bioactivity of the identified compounds. Some of these compounds have been reported previously to occur in plants of the same genus, such as *S. verticillate, S. articularis, S. exilis*, and *S. hispida* [6, 12, 13]*.*

**Fig. 3:**
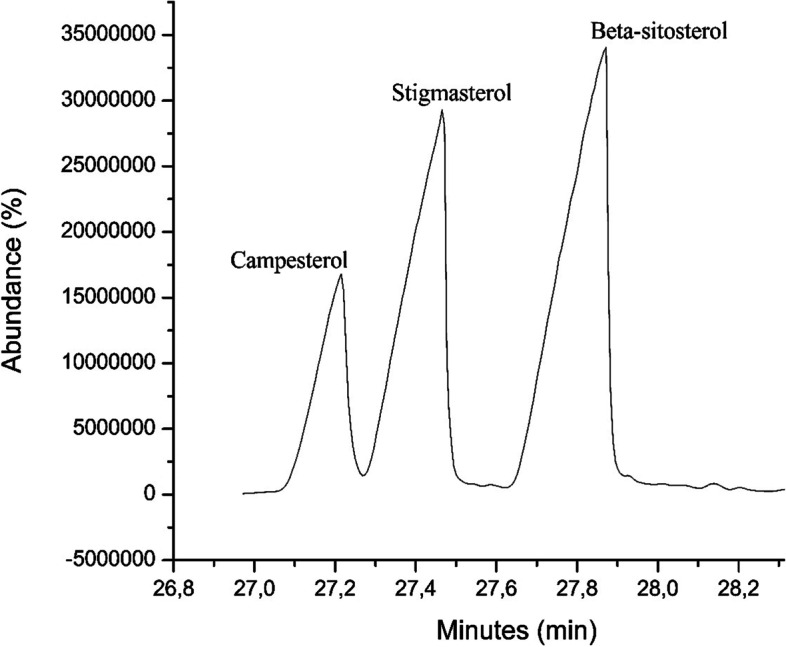
GC–MS profile of subfraction E-6–3 containing compounds **7**, **8** and **9**

**Fig. 4:**
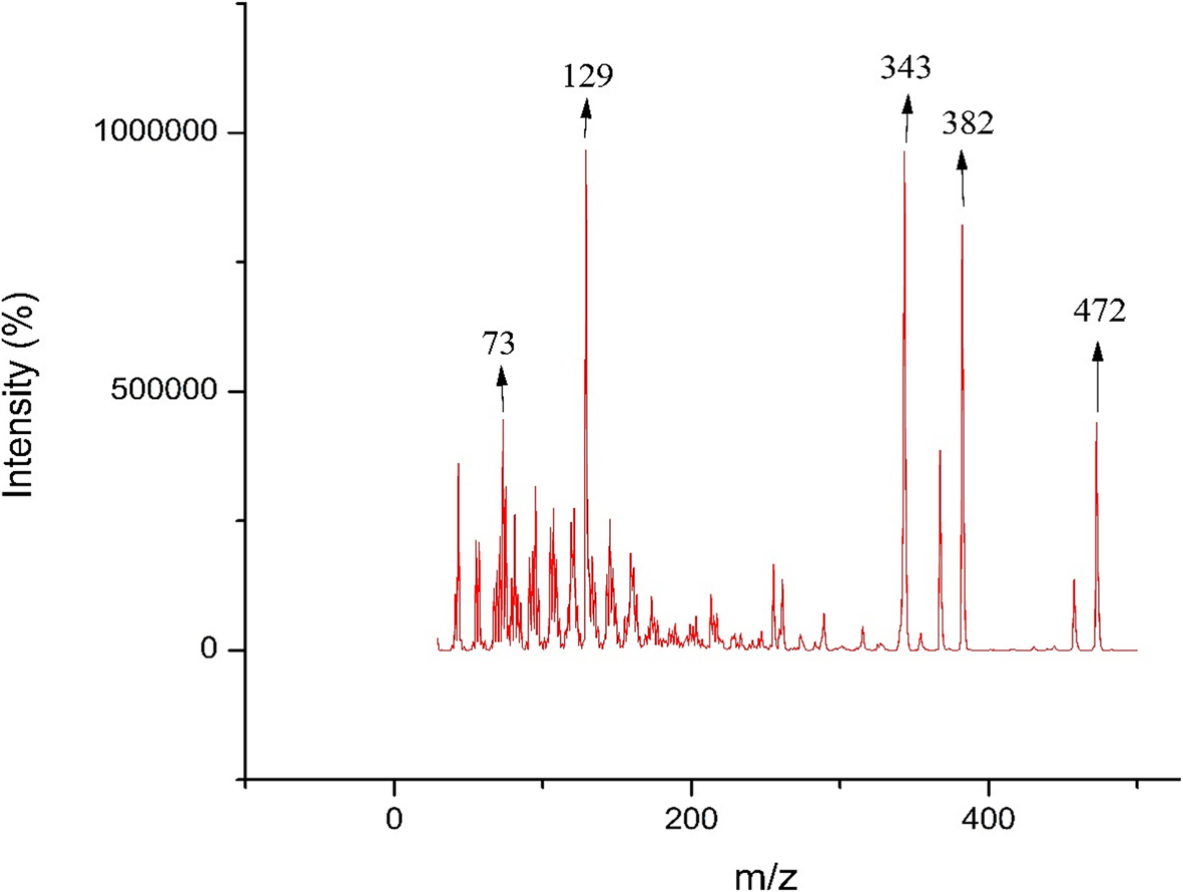
Mass chromatogram of compound **9**

GC–MS analysis of subfraction E-6–3 led to the identification of compound **9**, campesterol. The GC–MS data were compared with Wiley10 and the National Institute of Standards and Technology (NIST17) mass spectral libraries. The GC–MS spectrum of subfraction E-6–3 showed three peaks at RT (min); 27.21, 27.45, and 27.86 (Fig. 3). Analysis of the peak signals showed a molecular ion at; *m/z* 472 for campesterol (**9**) at 27.20 min, *m/z* 484 for stigmasterol (**8**) at 27.45 min, and *m/z* 486 for β-sitosterol (**7**). Characteristic fragment ions at *m/z* 382 for campesterol were observed (Fig. 4) while fragment ions at *m/z* 394 and 396, typical of stigmasterol and β-sitosterol respectively, were linked to the peaks at 27.45 and 27.86 min. All the peaks showed a molecular ion peak at *m/z* 129 which is a characteristic fragment of this phytosterol group [50].

### Antibacterial and antifungal activity of the isolated compounds

**Fig. 5:**
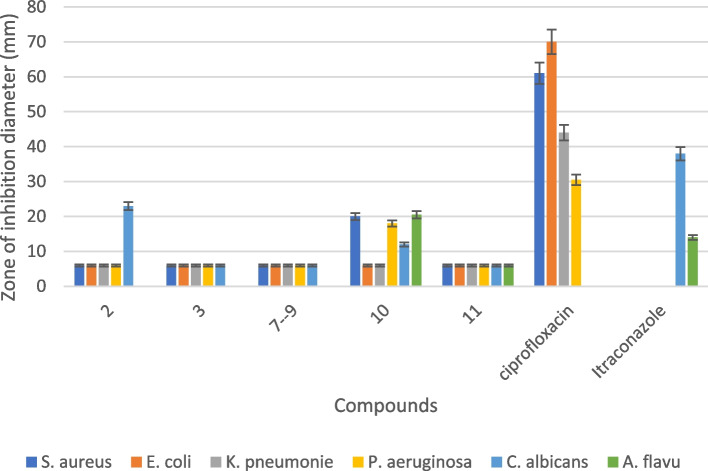
Diameter of inhibition zones for compounds (**2**, **3**, **7**–**11**) against bacterial/fungal strains

The antibacterial and antifungal activity of isolated compounds **2**, **3**, **7**, **8**, **9**, **10**, and **11** were examined against bacterial (*S. aureus, E. coli, K. pneumoniae,* and *P. aeruginosa*) and fungal (*C. albicans* and *A. flavus*) strains as shown in Fig. 5. Compound **10** showed activity against *S. aureus* (20.0 ± 0.1 mm)*, P. aeruginosa* (18.0 ± 0.1 mm)*, C. albicans* (12.0 ± 0.1 mm), and *A. flavus* (20.5 ± 0.3 mm)*.* Compound **2** showed activity against *C. albicans* (23.0 ± 0.1 mm). The data indicated that compound **10** displayed a wide degree of antibacterial and antifungal activity on the different tested micro-organisms. The other tested compounds did not show any activity against the tested bacterial and fungal strains. The quantity of compounds **1**, **4**, **5**, and **6** was only sufficient for spectroscopic analysis, but not for bioactivity testing.

### Antioxidant activity of the isolated compounds by DPPH (free radical scavenging) activity

Compounds **2** and **3** showed a good radical scavenging activity of 83.87 and 58.58% respectively. Compound **2** showed the highest radical scavenging activity among the extracted compounds tested (IC_50_ = 64.81 µg/ml). Ascorbic acid (IC_50_ = 2.59 × 10^–16^ µg/ml) was used as a positive control to determine the effectiveness of the extract in scavenging the free radicals*.* Compounds **7**, **8**, **9**, **10**, and **11** were only used for antimicrobial analysis.

### Sun protection potential of isolated compounds **2**, **3**, and **7**—**11**

**Fig. 6:**
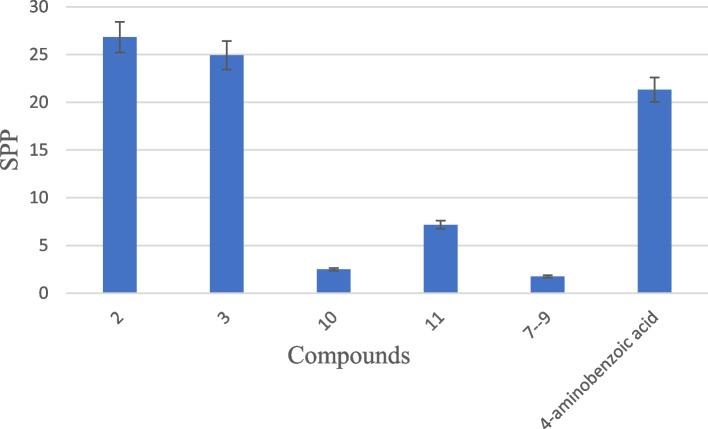
Sun protection potential of the isolated compounds **2**, **3**, and **7**–**11**

The sun protection potential of the isolated compounds is as shown in Fig. 6. Para-aminobenzoic acid (standard) was used to determine the effectiveness of the extract in protecting the skin against UV light. Compounds 2 (26.83 ± 0.27) and 3 (24.92 ± 0.31) showed a good ability to protect the skin against ultraviolet (UV) light.

## Discussion

**Fig. 7:**
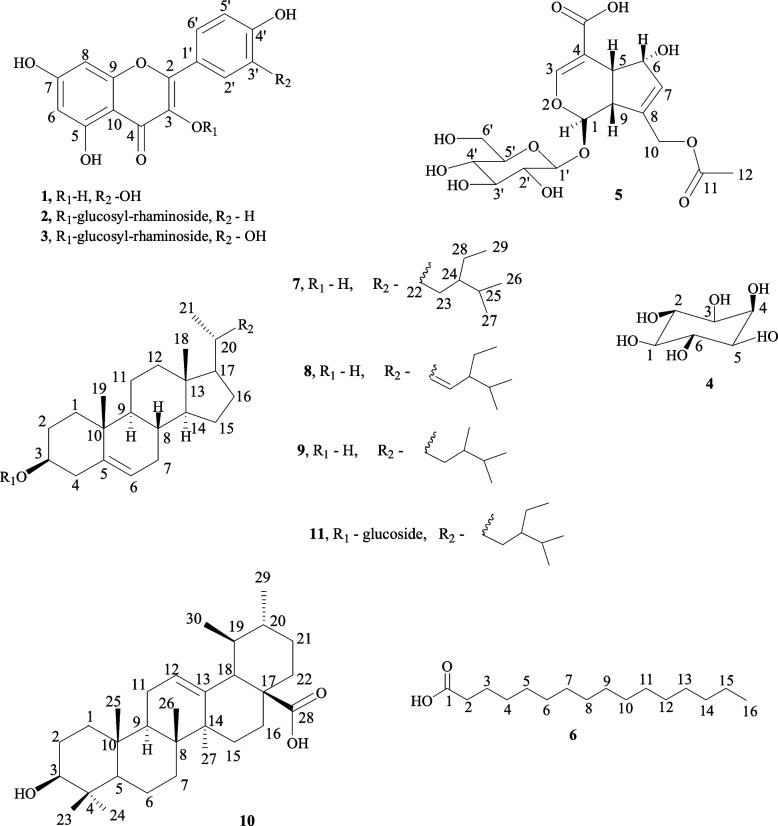
Chemical structures of compounds **1**–**11** isolated from *S. princeae*

Phytochemical analysis of the MeOH extract of *S. princeae* yielded three flavonoids (**1**–**3**), a monoterpene (**4**), an iridoid (**5**) characteristic of the family Rubiaceae [51], and an essential oil (**6**). Flavonoids (**2**, **3**) were the phytochemicals identified also in the aqueous extract. Triterpenoids (**7**–**11**) were the major phytochemicals in the EtOAc extract [1]. Figure 7 shows the chemical structures of the isolated compounds. This is the first report of the isolated active compounds from the aerial parts *S. princeae*. From Fig. 5, compounds **2** and **10** showed potential as antibacterial and antifungal agents. This agrees with a previous report, in which compound (**10**) from *Sambucus australis* has been reported to exhibit antibacterial activity against *S. aureus,* and *P. aeruginosa* [22, 43]*.* According to Namukobe et al. (2021), the EtOAc extract of *S. princeae* did not have any antibacterial potential. In this study, it was noticed that compound **10** which showed a good antibacterial and antifungal activity was isolated from the EtOAc extract.

Compounds **2**, and **10** demonstrated efficacy against *C. albicans*, *S. aureus* and *P. aeruginosa* strains. Thus, they could be used as antibacterial and antifungal agents. Antimicrobial flavonoids have multiple cellular targets and form complexes with proteins through nonspecific forces such as hydrogen bonding, hydrophobic effects, and covalent bond formation [52]. Thus, their mode of action (Table 1) may be related to their ability to inactivate microbial adhesins, enzymes, and cell envelope transport proteins [53, 54].

**Table 1:** Chemical identification numbers (CID) and summary of the mode of action of compounds **1**–**11**

Compound	Molecular formula	CID	Mode of action
**1**	C_15_H_10_O_7_	5280343	Compounds 1, 2 and 3 form complexes with proteins through nonspecific forces such as hydrogen donation [52, 55]
**2**	C_27_H_30_O_15_	5318767	
**3**	C_27_H_30_O_16_	5280805	
**4**	C_6_H_12_O_6_	892	Chelation of ferric ions and suppression of hydroxyl radicals [56]
**5**	C_18_H_24_O_12_	11968867	Suppresses NF-κB and MAPK Signaling Pathways in LPS-Induced RAW 264.7 Macrophages [57]
**6**	C_16_H_32_O_2_	985	Regulates cell proliferation by induced retinoic acid receptors [58]
**7**	C_29_H_50_O	222284	Reduces the phosphorylation of nuclear factor-kB p65 by binding U937 cells to TNF-a-stimulated HAEC [59]
**8**	C_29_H_48_O	5280794	Bonds with glucocorticoid receptors to induce the production of prostaglandins and other pro-inflammatory mediators [60]
**9**	C_28_H_48_O	173183	A secondary massager in the colon (HT29), breast (MCF7) or prostate (LNCap) cancer cells that activates ceramide metabolism [61]
**10**	C_30_H_48_O_3_	64945	Stimulates the nuclear translocation of glucocorticoid receptors [62]
**11**	C_35_H_60_O_6_	296119	Reduces the phosphorylation of nuclear factor-kB p65 by binding U937 cells to TNF-a-stimulated HAEC [59]

The identified compounds could explain the use of the plant in the treatment of skin infections and adjuvant effects in other diseases such as cancer or diabetes. From Table 2, both compounds **2** and **3** showed good antioxidant activity (IC_50_ = 64.81 and 666.85 µg/ml) indicating that the antioxidant activity of *S. princeae* methanol extract with IC_50_ = 61.26 µg/ml [11] was due to the presence of these compounds. By their antioxidant activity, the compounds could serve radical scavengers [63]. The antioxidant activity of flavonoids depends on the arrangement of functional groups in the aromatic structure. The configuration, substitution pattern, and total number of hydroxyl groups substantially influence the antioxidant activity. The B ring hydroxyl configuration is the most significant determinant of antioxidant activity because it can donate hydrogen and an electron to hydroxyl, peroxyl, and peroxynitrite radicals, in turn giving rise to a relatively stable flavonoid radical [52, 64].

**Table 2:** DPPH percentage scavenging activity of the compounds **2** and **3**

Compounds	Percentage scavenging activity (%), IC_50_ (µg/ml) in brackets)
Compound** 2**	83.87 ± 0.01 (64.81)
Compound** 3**	58.58 ± 0.02 (666.85)
**Ascorbic acid**	95.90 ± 0.05 (2.59 × 10^–16^)

**2**, kaempferol-3-*O*-rutinoside; **3**, rutin; IC_50,_ concentration of the compound required to scavenge DPPH by 50%

Sunscreens are chemicals that absorb UV rays protecting the skin from damaging solar radiation. [65]. In Fig. 6, compounds **2** and **3** showed high a level of against UV light compared to the standard para-aminobenzoic acid. The other compounds only exhibited a low level of protection against UV light. The recorded sun protection potential of the isolated compounds was better than that of crude methanolic and aqueous extract of *S. princeae* [11]. Solar ultraviolet radiation is made up of UV-C (200–280 nm), UV-B (280–320 nm), and UV-A (320–400 nm) [65]. UV-C is filtered out by the ozone layer and the most biologically damaging radiation, UV-B, and UV-A radiation are responsible for inducing skin cancer. The use of skin care products supplemented with several effective sunscreen agents may be an effective approach for reducing UV-B generated reactive oxygen spices as well as mediated photo-aging [66].

Some of the isolated compounds have been previously reported to possess variable biological activities with different mode of action as summaries in Table 1. Compounds **1**, **2**, and **3** have antibacterial, antifungal, antioxidant, and sun protection potential [52, 67–69]. Compound **5** has been reported to exhibit antioxidant activity, one of the studied has reported a good renal interstitial fibrosis effects, characterized by the accumulation of excess extracellular matrix and renal tissue damage in the kidney [57, 70]. However, its antibacterial potential is still lacking. Similar compounds such as asperulosidic acid methyl ester, have been reported to possess good antifungal activity against *C. albicans* (8.33 mm zone of inhibition diameter) [71]. Compound **4** is a major form through which plants store phosphorus [72, 73] and has been reported as a metabolic mediator during the transcription of stimuli-responsive genes in stress response and hormones. It is used in treating mood disorders but no studies have been carried out to investigate its antioxidant, sun protection and antibacterial potential [56, 74]. Compounds **7**, **8**, **9** and **11** have antibacterial and antioxidant activity [75–77].

## Conclusion

This study provides the scientific basis for the ethnopharmacological use of *S. princeae* for the treatment of skin infections, with 11 bioactive compounds having been isolated from the extracts and unambiguously identified. Compound **2**, kaempferol-3-*O*-rutinoside has antifungal activity against *C. albicans.* Compound **10**, ursolic acid shows various antibacterial and antifungal activities against *S. aureus, P. aeruginosa, C. albicans* and *A. flavus*. Therefore, these compounds explain the effects observed and used in traditional medicine. They should be considered in drug formulations and be further evaluated for their cytotoxicity, to establish their mode of action, sensitivity, and selectivity. In future work, we will address further in-dept analysis of the compounds contained in the *n-*hexane and aqueous extracts of *Spermacoce princeae*.

## Data Availability

The datasets used and/or analyzed during the current study are available from the corresponding author on reasonable request and Mendeley data repository DOI.10.17632/x2y89bdcj8.1

## Abbreviations

DCM: Dichloromethane
EtOAc: Ethyl acetate
MeOH: Methanol
Hex: *n-*Hexane
DMSO: Dimethyl sulfoxide
UV: Ultra Violet
VIS: Visible
NMR: Nuclear Magnetic Resonance
Q-ToF: Quadrupole time- of-flight
MS: Mass spectroscopy
GC: Gas chromatography
FID: Flame Ionization Detector
MHz: Megahertz
